# Centromeric Transcription: A Conserved Swiss-Army Knife

**DOI:** 10.3390/genes11080911

**Published:** 2020-08-09

**Authors:** Ganesan Arunkumar, Daniël P. Melters

**Affiliations:** Chromatin Structure and Epigenetic Mechanisms, Laboratory of Receptor Biology and Gene Expression, Center for Cancer Research, NCI, NIH, Bethesda, MD 20892, USA; arunkumar.ganesan@nih.gov

**Keywords:** centromere, transcription, non-coding RNAs, nucleosomes, chromatin, epigenetics

## Abstract

In most species, the centromere is comprised of repetitive DNA sequences, which rapidly evolve. Paradoxically, centromeres fulfill an essential function during mitosis, as they are the chromosomal sites wherein, through the kinetochore, the mitotic spindles bind. It is now generally accepted that centromeres are transcribed, and that such transcription is associated with a broad range of functions. More than a decade of work on this topic has shown that centromeric transcripts are found across the eukaryotic tree and associate with heterochromatin formation, chromatin structure, kinetochore structure, centromeric protein loading, and inner centromere signaling. In this review, we discuss the conservation of small and long non-coding centromeric RNAs, their associations with various centromeric functions, and their potential roles in disease.

## 1. Introduction

Centromeres are essential for ensuring accurate chromosome segregation in eukaryotes. Centromeric chromatin is characterized by the enrichment of nucleosomes containing the centromere-specific histone H3 variant CENP-A/CENH3, which in turn, directly and indirectly, recruit kinetochore components. Despite their essential function, these centromeric and kinetochore proteins are fast-evolving [1,2,3]. This rapid evolution is also a characteristic of centromeric DNAs [4,5], which are highly repetitive in nature. This discrepancy between the essential function of centromeres and the rapid evolution of its components is known as the centromere paradox [5].

Despite the variation in centromere structure and sequence, a common theme has recently emerged. Whether a point centromere or a regional centromere, centromeric transcription is critical for centromere function. Interestingly, centromeric transcripts (cenRNAs) are found in two forms: long non-coding RNAs (lncRNAs) (>200 nt) and small RNAs (<200 nt). Indeed, the rapid evolution of centromeric DNA is mirrored by the rapid evolution of lncRNAs [6,7]. In general, lncRNAs can be processed based on their nuclear localization [8,9], but they can also be processed into siRNAs by dicer [9,10]. As the functions of more lncRNAs are being dissected, their roles in modulating mRNA cleavage, translational repression, and regulation of alternative splicing, have become apparent [8,9,11]. Although many lncRNAs are fast evolving, several lncRNAs are conserved at both the sequence and synteny (physical co-localization on genetic locus) levels [6,7], suggesting that some lncRNAs might be conserved at the functional level across species. Surprisingly, a recent study [12] showed that a conserved lncRNA expressed in human and mouse embryonic stem cells displayed surprisingly divergent subcellular localization and functions. The human version of the lncRNA was spliced more often, resulting in preferential cytoplasmic localization, whereas the mouse version was exclusively nuclear [12].

Although centromeres are transcribed, it remains unclear what features of these transcripts drive their associated functions. Centromeric DNA sequences evolve rapidly and only show limited conservation, even among closely related species [4]. In addition, centromeres are known to reposition on chromosomes [13]. This creates a scenario wherein centromeric lncRNAs are conserved based on their function rather than on their sequence and synteny. In this review, we highlight recent advances in our understanding of centromeric transcription, how common centromeric transcription is, how transcripts may impact centromere biology, and how aberrant centromeric transcription contributes to disease.

## 2. Centromeric Transcription

**Figure 1:**
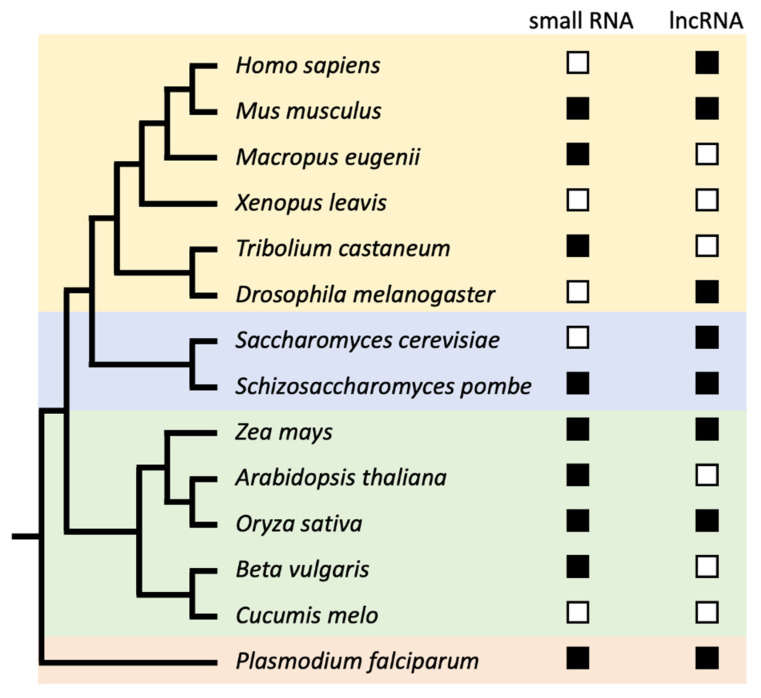
Cladogram showing which types of transcripts have been found in which species. The presence of centromeric small RNAs (<200 nt) and centromeric lncRNA (>200 nt) is shown behind each species described in Table 1. The colors yellow, blue, green, and red represent the Animalia, Fungi, Plantae, and Chromista kingdoms, respectively.

RNA polymerase 2 (RNAP2) is capable of transcribing most DNA sequences without strong sequence-specificity [14,15]. Consequently, as long as transcriptional initiation and elongation occur, RNAP2 will transcribe most underlying DNA [14,16,17,18]. Sequence-specific factors, such as TBP, help direct where the pre-initiation complex is assembled and thus where transcription occurs [16,18]. In contrast, for highly repetitive sequences, such as centromeric DNA, no strong promoter activity has been reported [19,20]. Nevertheless, centromeric transcripts have been observed across a broad range of species (Table 1, Figure 1). The transcripts identified so far fall into two main categories: long non-coding RNAs (>200 nt) and small RNAs (<200 nt).

### 2.1. Centromeric Long Non-Coding Transcripts

The first reported centromeric transcripts came from mouse satellite DNA in the late 1960s [36,37], but it took several more decades before the functional implication of centromeric transcription was appreciated. Today, centromeric transcripts have been observed in Apicomplexa, plants, fungi, and animals (Table 1, Figure 1). Their sequences and transcript lengths remain to be determined for most species. A fundamental problem is that most centromeres do not appear to have well-defined lncRNA genes with distinctive genic features such as promoters, splice sites, and polyadenylation signals. Consequently, with short-read sequencing technologies, although they effective at assessing the transcription and splicing of annotated genes, it is technically difficult to assemble de novo transcripts from centromeric RNAs [79,80]. An alternative method to assess the length and abundance of centromeric transcripts is to perform a Northern blot from isolated total RNAs. Indeed, such efforts have been made in various species with varying results (Figure 1). In human cell lines, one study used a consensus α-satellite probe and found that human centromeric transcripts are ≈1.3 kb in length [21]. However, another study used probes specific to centromeric D17Z1, D17Z1-B, and DXZ1 arrays, and observed a smear ranging from 0.3 to 2 kb [22]. In mouse cell lines different sizes of satellite RNA (0.12–4 kb) were found. Curiously, the 120-nt minor satellite transcripts were sensitive to different growing conditions [35]. This shows that centromeric transcription can be regulated by external stimuli. Uncovering the functional consequences of altering the transcriptional output of centromeres is important for understanding how centromeric transcription is related to disease.

In fission yeast, the central centromere core *Cnt1* produces transcripts of ≈0.5 kb in length [57]. Interestingly, different RNA-processing and kinetochore mutants changed the abundance of this transcript [57]. Many plants have retrotransposons at their centromeres, which are expressed (Table 1). In rice, in addition to the smear of 4–15 kb, a distinct band at ≈3.1 kb was observed for various centromeric retrotransposons [74]. In maize, the centromere-specific retrotransposon CRM-derived transcripts are 40–900 nt in length [70]. Whereas most species have a regional centromere that is commonly comprised of tandem repeat arrays and transposable elements, budding yeast has a point centromere [81]. Despite the seemingly stark differences in DNA sequence organization and DNA binding capabilities, a pivotal study demonstrated that budding yeast centromeres are also transcribed into lncRNAs and that these lncRNAs are important for centromere homeostasis [54,55]. That a broad range of species produce lncRNAs from centromeric DNA suggests that centromeric lncRNAs serve a functional role in centromere biology. A key missing part of the puzzle is whether centromeric transcription also occurs in holocentric species. Work on the nematode *Caenorhabditis elegans* [82] and lepidopteran *Bombyx mori* [83] indicate anti-correlations between active transcription and the localizations of CENP-A and CENP-T chromatin, respectively. These data were obtained in asynchronized cells, which might not reveal low-level transcription at centromeric chromatin. It will be interesting to learn whether holocentric transcription indeed does occur, despite the complicated nature of studying centromeres with a diffuse phenotype.

### 2.2. Centromeric Small RNAs

In addition to lncRNAs, small RNAs (<200 nt in length) from centromeric DNA have also been found across a broad range of species (Table 1, Figure 1). Indeed, centromeric small RNAs were first isolated from *Arabidopsis* [73], after strong clues came from fission yeast studies that showed that deletion of components of the RNAi machinery resulted in aberrant accumulation of pericentromeric transcripts [64,65]. The centromeric structure of fission yeast is distinctly different from that of most plants and animals. The centromere core, where CENP-A nucleosomes are localized, is flanked by pericentric inverted repeats [84]. These repeats are transcribed and essential for pericentric heterochromatin formation [85,86,87]. This clear-cut demarcation between the functional centromere and pericentromere is much fuzzier in repeat-rich regional centromeres. Nevertheless, a similar increase in the abundance of centromeric small RNAs was observed in *Arabidopsis* mutants of the RNAi machinery and histone deacetylases [73]. Shortly after the identification of centromeric small RNAs in *Arabidopsis*, small RNAs were isolated from non-mutant rice plants [75]. In addition, centromeric small RNAs have also been identified in the *Plasmodium* parasite [78], sugar beet [76], red flour beetle [50], and Tammar wallaby [45]. These findings provide further evidence that small RNAs are produced from centromeric DNA across kingdoms and that these small RNAs are strongly associated with the RNAi machinery.

### 2.3. Post-Transcriptional Processing of Centromeric Transcripts

It has long been appreciated that more RNA is produced than is strictly needed to maintain all cellular functions. In addition to pre-mRNA being processed into a mature mRNA, many RNAs are actively degraded [88,89]. Although genes can be embedded in centromeric chromatin [90], repetitive centromeric DNA is not known to encode proteins. Nevertheless, polyadenylation isolation protocols consistently detect centromeric transcripts across kingdoms (Table 1), providing evidence that cenRNAs can be processed like lncRNAs and mRNAs. These two latter groups of transcripts also tend to undergo splicing events to produce mature transcripts [91,92]. Indeed, centromeric RNAs are associated with splicing factors (Table 1). In the case of fission yeast, the pericentric non-coding *dg* RNA contains an intron that plays an important role in the recruitment of the RNAi-machinery needed for pericentric heterochromatin formation [63]. Human α-satellite has been found to associate with the RNA helicase DHX38 [32], which is thought to be important for pre-mRNA splicing [93]. Although the data are sparse thus far, they do provide tantalizing hints that centromeric RNAs interact with various RNA processing components. Whether splicing itself is an important maturation step for cenRNAs or whether the splicing machinery serves as a physical link with other nuclear components remains to be determined.

In several species, both centromeric lncRNA and small RNAs have been found (Table 1). Thus far though, in most species, only one of the two RNAs has been identified. One technical reason for this may be that different RNA isolation methods do not isolate all types of RNA equally, especially if the RNA types are not equally abundant or stable [94]. Identification of centromeric transcripts depends on knowing the sequence composition of the actively transcribing centromere in each species. Understanding the relationship between centromeric lncRNAs and small RNA production is important. Recently, the sequence of the fruit fly centromere was deciphered [95] and this has opened up a new avenue to study the evolution of centromeric transcription in a highly tractable system. Indeed, in *Drosophila* species centromeric DNA is highly repetitive and fast-evolving [96]. In other words, it will be of interest to understand the evolutionary conservation of the production of both centromeric lncRNAs and small RNAs. Furthermore, uncovering when and how centromeric transcripts are processed and with which biological function this processing is associated is an exciting avenue for future research.

## 3. Functions of Centromeric Transcription

**Figure 2:**
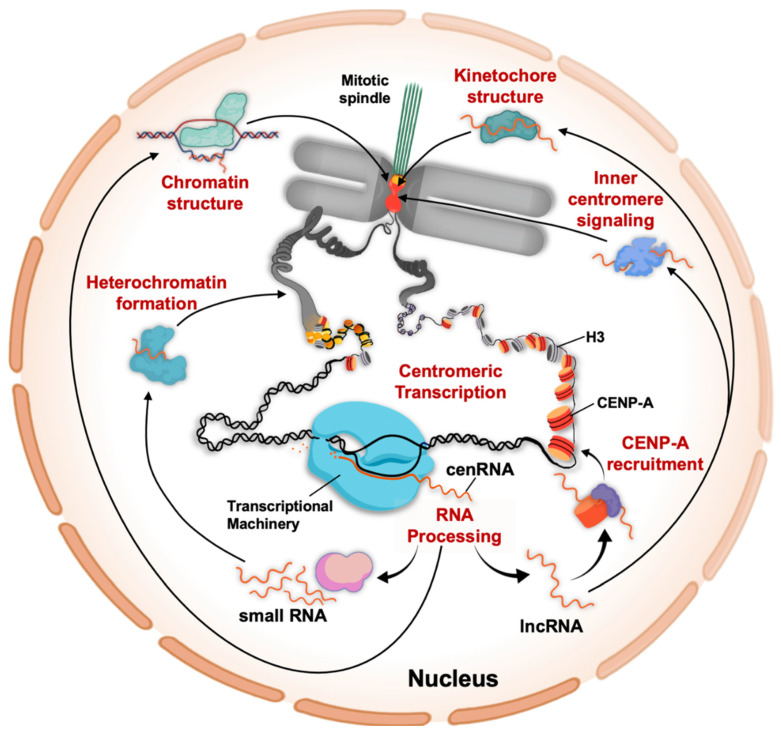
Biological functions of centromeric transcripts. The centromere-specific nucleosomes are distributed in a species-specific manner. For instance, in fission yeast, CENP-A^Cnp1^ nucleosomes are restricted to the centromere core, whereas in humans CENP-A chromatin is interspersed with H3 chromatin at the centromere. Despite these different distribution patterns of CENP-A chromatin, centromeric transcription has been observed in a broad range of species. Centromeric transcripts are processed and are found as either lncRNAs or small RNAs. Through the argonaut/dicer machinery, centromeric small RNAs facilitate the pericentric heterochromatin formation. Centromeric chromatin structure can be modulated through DNA-RNA hybrids as well as through CENP-B, both of which associate with centromeric transcripts. Centromeric lncRNAs facilitate recruitment and loading of new CENP-A nucleosomes, whereas the kinetochore component CENP-C pulls down centromeric transcripts as well, contributing to kinetochore formation and structure. Finally, Aurora B signaling at the inner centromere is critical for faithful chromosome segregation, and centromeric transcription is important for this process.

A logical prediction for the existence of the two classes of centromeric RNAs is that the lncRNA is the precursor of the small RNAs to ultimately produce pericentromeric heterochromatin (Figure 2). Yet non-centromeric, long non-coding RNAs have been implicated in a much broader range of cellular functions, including chromatin architecture, chromatin remodeling, transcriptional regulation, formation of nuclear bodies, and translational regulation [8,97]. Overall, lncRNA can function as a guide, scaffold, decoy, or signal [6]. In fact, well-known chromatin binding factors, such as CTCF [98] and the polycomb complex [99], not only bind distinct DNA motifs but are also functionally associated with RNA. This raises the question, what is the full breadth of the functional implications of centromeric transcription?

### 3.1. Recruitment and Loading of CENP-A 

During every cell division, the total number of centromeric CENP-A nucleosomes is halved [100]. To guarantee continued faithful chromosome segregation over many cell cycles, the number of centromeric CENP-A nucleosomes must be maintained to guarantee that the kinetochore can be formed. Thus, the centromeric CENP-A nucleosome pool must be replenished with new CENP-A nucleosomes. Indeed, CENP-A nucleosomes are loaded on centromeric chromatin in a cell cycle-specific manner [54,100,101,102,103]. In the last several years it has been shown that centromeric transcription plays critical roles in various aspects of loading of new CENP-A nucleosomes. The first clues came from studies in fission yeast; various mutants of the RNAP2 machinery, including transcription initiation and elongation factors, showed reduced levels of CENP-A^Cnp1^ [57,104]. Direct evidence came from work in mammalian cell lines and *Xenopus* oocyte extracts where knock-down of centromeric transcripts resulted in reduced CENP-A levels at the centromere [21,24,25,26,33,48]. Indeed, recent work in mammalian and fruit fly cell lines showed that chemical inhibition of activated RNAP2 resulted in the loss of centromeric CENP-A^CID^ chromatin [21,52]; and the elongation factor Spt6 facilitates maintenance of centromeric CENP-A^CID^ [105]. These lines of investigation strongly hint at a distinct role for both the act of transcription and the production of an RNA species in the loading of CENP-A.

It is not very likely that new CENP-A is loaded exclusively on naked DNA, rather than on chromatin. Indeed, in human and fruit fly cell lines, active transcription is needed to remove so-called placeholder H3.3 nucleosomes prior to new CENP-A loading at the centromere [53,105,106]. Similarly, in fission yeast, H3 nucleosomes function as placeholder nucleosomes [61]. None of these experiments addressed how CENP-A:H4 is recruited to the centromere. In many species, a dedicated CENP-A chaperone has been identified that performs this function. Human cells use HJURP [107,108] and RbAp48 [109], fruit flies use CAL1 [110], fission and budding yeast uses SCM3 [111,112,113,114], the holocentric roundworm *Caenorhabditis elegans* uses RbAp46/48^LIN-53^ [115], and *Arabidopsis thaliana* uses NASP^SIM3^ [116]. Although knock-down of fruit fly 359-bp satellite RNA resulted in reduced levels of CAL1 at the centromere [51], HJURP has been shown to directly interact with α-satellite lncRNAs [21]. Additionally, soluble pre-assembled HJURP/CENP-A complexes were observed, and knock-down of these α-satellite lncRNAs resulted in reduced levels of CENP-A and HJURP at the centromere [21]. Altogether, a picture emerges wherein centromeric transcription plays multiple active roles in both recruitment and incorporation of new CENP-A nucleosomes, including the eviction of placeholder nucleosomes. This begs the question: What are the precise roles of each step of transcription of centromeric DNA and subsequent post-transcriptional RNA processing in establishing and maintaining centromere identity?

### 3.2. Recruitment of CENP-C

Recent work showed that knock-down of CENP-C resulted in increased centromeric transcription [117], whereas knock-down of centromeric transcripts resulted in increased levels of centromeric CENP-C [31]. These data suggest that there is a functional interaction between CENP-C and centromeric transcription. The question remains as to whether this is an indirect effect, driven by CENP-A, or a direct effect. A direct association between CENP-C and centromeric transcripts is possible, as CENP-C has extensive nucleic acid binding activity [24,69,118,119], and both budding yeast and human CENP-C associates with AT-rich DNA [118,119]. Interestingly, in vitro, a 24-nt single-stranded centromeric RNA facilitates the binding of maize CENP-C to centromeric DNA [69]. Outside of the centromere, RNA–DNA triplexes are stabilized by nucleosomes in vitro; and in human cells, these RNA–DNA triplex structures were enriched at active regulatory sites [120]. This raises the possibility that RNA has a pivotal function in stabilizing CENP-C at the centromere, either in a CENP-A-dependent or CENP-A-independent manner. It will be interesting to dissect how centromeric RNA contributes to CENP-C localization and stabilization on centromeric DNA in other eukaryotic species.

### 3.3. Centromeric RNA, CENP-B, and Centromeric Chromatin Structure

Interestingly, the DNA binding centromeric protein CENP-B has also been found to associate with centromeric RNAs (Table 1). CENP-B binds to the 17-bp CENP-B box sequence [121], which is found in a subpopulation of α-satellite DNA, which can be occupied by either CENP-A or H3 nucleosomes [122,123,124]. In addition, CENP-B binds at the DNA entry site of CENP-A nucleosomes, helping to phase CENP-A nucleosomes on centromeric DNA [125]. On α-satellite arrays on human artificial chromosomes, the presence of CENP-B is critical for the recruitment of CENP-A nucleosomes [126,127]. Similarly, when endogenous CENP-A is degraded using the auxin-inducible system, CENP-B still partially allows centromeres to form a functional kinetochore [128]. Interestingly, a recent study showed that CENP-B serves as a “beacon” for H3.3 incorporation [129]. Furthermore, in a recent fluorescence microscopy-based interaction-trap assay, components of both heterochromatin (SUV39H1 and HP1) and open chromatin (including histone methyltransferases ASH1L and NSD1) were recruited by CENP-B [130]. These results point towards a role for CENP-B in organizing centromeric chromatin structures to be competent for both new CENP-A loading and heterochromatin formation. Nevertheless, it remains poorly understood whether or not, and if so how, centromeric transcription facilitates CENP-B’s role in chromatin organization. Therefore, understanding whether or not, and if so how, centromeric transcripts associated with CENP-B help to distinguish between the formation of these two mutually exclusive chromatin domains is important. From a chromatin organizational perspective, CENP-B can potentially cross-link neighboring chromatin fibers through its dimerization domain [131]. In addition, RNA–DNA triplexes [132], such as R-loops, have also been observed at centromeres, and these R-loops were associated with the ATR kinase, which is important in safeguarding genome stability [133]. In maize, circular RNA derived from centromeric CRM retroelements potentially associated with two R-loops, and thereby formatted chromatin loops. Knock-down of these circular RNAs resulted in the loss of chromatin loops and reduced levels of CENP-A^CENH3^ [72]. These latter studies imply that R-loops might be involved in organizing local chromatin structures which might contribute to the regulation of ATM/ATR kinase activity. Thus, understanding how centromeric chromatin conformation contributes to both centromere organization and centromere maintenance is an important question to explore.

### 3.4. Inner Centromere Signaling

In addition to the functions described above, centromeric transcripts have another critical role in centromere biology. Prior to anaphase, the mitotic checkpoint must be met, guaranteeing that all chromosomes are properly oriented and attached to the mitotic spindles. At the chromatin region (inner centromere) between the inter-sister chromatids, the chromosomal passenger complex (CPC) accumulates [134] and senses and responds to the pulling forces generated at the kinetochores [135,136]. The CPC is comprised of the mitotic kinase Aurora B, INCENP, survivin, and borealin [134]. Recently, CPC was reported to form coacervates, which are thought to be functionally important [137]. Interestingly, in various vertebrate species, CPC components have been shown to pull down centromeric transcripts (Table 1). In particular, when Aurora B is bound to centromeric RNA, it regulates both its activity and its localization [47]. Shugoshin (SGO1) is thought to protect centromeric cohesion from cleavage during prophase [138], but SGO1 also brings RNAP2 to the centromere [29] and indeed associates with centromeric RNA as well [29]. Mitotic transcription specifically is important for the spatiotemporal functioning of CPC at the inner centromeres [47]. Interestingly, using a nucleosome affinity library a recent study showed that the CPC interacts with the nucleosome acid patch [139] and the acid patch is known to modulate higher-order chromatin structure [140]. Thus, how centromere chromatin structures dictate the recruitment of SGO1 and CPC; and how CPC modifies the centromere chromatin to facilitate mitotic progression are exciting avenues for future studies.

### 3.5. Pericentromeric Heterochromatin

In fission yeast, the importance of pericentromeric transcription for heterochromatin formation has been extensively studied (reviewed here [86]). Beyond fission yeast, only a few studies have addressed directly whether centromeric transcription plays an important role in pericentromeric heterochromatin formation [23,43,141]. Why are CENP-A nucleosomes deposited where they are and not somewhere else on the chromosome? In addition, ectopic CENP-A^CID^ prefers to seed at euchromatin-heterochromatin boundaries [142]. This latter observation suggests that CENP-A chromatin prefer to be right next to a heterochromatin domain. If centromeric transcription can create a chromatin environment that is conducive to both heterochromatin formation and kinetochore establishment, it would be the ideal double-edged sword. Two recent studies in human and mouse cells [23,141] showed that centromeric RNAs are important for heterochromatin formation. In the paternal mouse pronucleus, pericentric transcripts suppress SUV39H2 activity, resulting in subsequent reduced H3K9me3 levels [141], potentially through the formation of RNA–DNA triplexes near nucleosomes [43]. In contrast, in human cells, SUV39H1 needs to bind α-satellite RNA to establish constitutive heterochromatin [23]. The latter scenario is more reminiscent of what is observed in fission yeast, wherein, through a dicer-mediated pathway, constitutive heterochromatin is established and maintained [86]. Indeed, in mouse cell lines, dicer has been associated with centromeric RNA [41]. It is therefore conceivable that centromeric small RNAs might have specific developmental roles in establishing where and when constitutive heterochromatin is formed. The make-up of the respective protein complexes with which centromere small RNAs associate might provide important clues.

## 4. Centromeric Transcription in Disease

Given the biological functions with which centromeric transcripts are thought to be associated, misregulation seems almost inevitable. Indeed, the misregulation of mouse minor satellite RNAs leads to impaired centromere function and defective chromosome segregation [35]. In addition, work in budding yeast has focused on how CENP-A^Cse4^ loading is limited to the centromere [143,144,145,146,147]. Whether the removal of ectopic CENP-A in non-Ascomycota species happens by the same mechanism with the same efficiency remains to be determined. The fact that centromeric DNA is not conserved strongly argues against a model wherein the sequence is the driving factor in the functional consequences of centromeric transcription. Thus, the question of whether transcription at non-centromeric loci can hijack centromere-like functions is at the heart of ectopic centromeric chromatin formation.

### 4.1. Ectopic CENP-A and Neocentromeres 

In many cancers, various centromere and kinetochore components are overexpressed, including CENP-A and its dedicated chaperone HJURP [148]. Overexpressed CENP-A has been found to not only associate with HJURP but also with the H3.3 chaperones DAXX/ARTX and HIRA [149,150,151,152], resulting in ectopic incorporation of CENP-A nucleosomes. One potential consequence of ectopic CENP-A chromatin sites is the formation of dicentric chromosomes, rendering the chromosome unstable [153]. Indeed, ectopic CENP-A sites promote the formation of ectopic kinetochores and subsequent mitotic defects [150]. Similarly, artificially overexpressed CENP-A^CID^ in Schneider S2 cells was also able to form functional ectopic kinetochores [142]. Similarly, in fission yeast [154] and budding yeast [155,156], ectopic localization of CENP-A occurs when CENP-A is overexpressed. In another experimental setup, ectopic CENP-A was found when the innate centromere was deleted [157].

These results give the impression that CENP-A readily goes ectopic, consistently posing the risk of creating dicentric chromosomes. One would expect that mechanisms have evolved to reduce this risk to a minimum without impacting the native centromere functions. In the case of the point centromere of budding yeast, which is thought to contain only of a single CENP-A^Cse4^ nucleosome [158], SUMOylation of N-terminal tail of CENP-A^Cse4^ is a driving force in preventing CENP-A^Cse4^ mislocalization [143,144,145,146,147]. In plants, active removal of CENP-A^Cse4^ has also been observed [159]. In contrast, a recent study in human cell lines suggested that ectopic CENP-A nucleosomes are removed during replication [160]. Nevertheless, in various cancers, ectopic CENP-A nucleosomes are readily detected [149,161]. This either means that, in the case of the human cells, replication has a limited capacity to correct ectopic localization of CENP-A nucleosomes, or an alternative mechanism is actively recruiting CENP-A to ectopic sites. As centromeric DNA evolves very quickly, it is unlikely that a strong sequence signal is driving the functional associations between centromeric transcripts and new CENP-A loading. Nevertheless, neocentromeres and ectopic CENP-A sites are not randomly distributed across the human genome [149], implying that certain loci have centromeric potential, whereas other loci do not. Studies on human artificial chromosomes showed that a chromatin environment containing a distinct set and distribution of histone modifications was important for CENP-A chromatin formation as well [162,163,164]. Using the marker chromosome mardel(10), L1 retrotransposon transcripts have been found to facilitate the formation of CENP-A chromatin [165]. Given these results, it is therefore tempting to speculate that some non-centromeric regions might have the capacity to mimic transcriptional conditions of the centromere. 

### 4.2. Centromeric Transcription in Cancer

What happens when centromeric transcription is misregulated? One may predict that when centromeric transcription is suppressed, essential centromeric functions during mitosis and CENP-A chromatin maintenance is impaired, resulting in either cell death or senescence. On the other hand, overexpression of centromeric transcripts may result in aberrant regulation of mitosis, resulting in genomic instability. Indeed, in various human cancers, α-satellite transcripts are overexpressed [166], and overexpression of these transcripts strongly correlates with a poor clinical prognosis [148]. In human and murine cell lines centromeric transcripts can accumulate as a result of DNA demethylation, heat shock, or the induction of apoptosis [167]. In these cases, and overexpression of centromeric RNA, genomic instability is observed [167,168], and genome instability is one of the hallmarks of cancer [169]. 

In human mammary epithelial cells derived from several patients, artificial overexpression of α-satellite sequences resulted in both hypomethylation of centromeric DNA and a significant increase in segregation errors, especially in chromosomes 8 and 20 [170]. Furthermore, in mouse and human breast tumors lacking BRCA1, satellite RNAs are highly expressed [171,172], as BRCA1 facilitates the monoubiquitination of histones associated with satellite DNA, thereby suppressing transcription [171]. When satellite RNAs are overexpressed the DNA damage response pathways are activated, inducing aneuploidy, which, in mice, is sufficient for tumor development in mammary glands [172]. Interestingly, some centromeric transcripts remained associated with distinct genomic loci, even throughout mitosis [173,174], promoting the localization of chromatin factors, such as PRC1 and MeCP2 [175]. Furthermore, centromeric transcripts have also been associated with repeat expansion [176]. Whether misregulation of centromeric transcription also facilitates the expansion of the pericentric HERV-K retrovirus [177] remains to be determined. In a recent study in fission yeast, mutations in RNAP2 or TFIIS^Tfs1^, a transcription factor that facilitates RNAP2 restarting after backtracking, increased the incidence of chromosomal rearrangements [178]. All in all, these studies hint at the possibility that genomic instability as a consequence of aberrant centromeric transcription is caused by a broad range of nuclear functions.

Thus, what are the functional consequences of overexpressing of centromeric transcripts that can be directly attributed to these transcripts? One clue how a non-coding RNA can impact nuclear organization and function comes from the overexpression of the unique lncRNA: ChRO1. ChRO1 expressed specifically during the terminal myoblast differentiation and is involved in the reorganization of constitutive heterochromatin [179]. Inhibition of ChRO1 leads to aberrant chromatin organization and mislocalization of histone marks and chromatin binding factors in murine cells [179]. In particular, ChRO1 mediates centromeric RNA accumulation at centromeric sites through DAXX/ARTX/H3.3 [179]. Interestingly, in fission yeast, a link between the maintenance and nuclear localization of pericentric heterochromatin and histone chaperone complexes was also observed [180]. 

As noted above, overexpressed CENP-A can associate with H3.3 chaperones [149,150,151,152]. Therefore, in many cancers, both CENP-A and centromeric transcripts are frequently overexpressed. Whether these two observations are dependent on each other or have a cumulative or even synergistic effect on tumor development is still an unanswered question. How the mislocalization of histone variants is driven by the expression of non-coding RNAs and how histone chaperone complexes are guided by non-coding RNAs remain of subjects of intense investigation. It is interesting to speculate that the role of histone chaperones go beyond assisting histone localization and nucleosomes dynamics [181]. Maybe, histone chaperones function as a critical factor contributing to chromatin domain formation, long-distance chromatin interactions, and even mechanosensing [182] by bringing together nucleosomes, non-coding RNAs, and other chromatin binding factors. Consequently, misregulation would lead to a broad range of chromatin effects, which are commonly observed in cancers.

## 5. Conclusions and Future Perspective

Centromeres are genetically different across species and they do not follow synteny [5]. In addition, frequent ectopic incorporation of CENP-A and the formation of neocentromeres [28,149,150] clearly indicate that centromere formation is not primarily driven by genetic factors, but rather a complex interplay between various epigenetic mechanisms. In most species [183], nucleosomes containing centromere-specific histone variants and their cognate binding partners form the proteinaceous basis of the kinetochore. Over the last few decades, it has become apparent that centromeric transcription is found across the eukaryotic tree (Figure 1). Although not every function has been shown to exist in every species studied so far (Table 1), it is reasonable to predict that these transcripts fulfill a multitude of roles (Figure 2), as if centromeric transcription were a swiss-army knife.

**Figure 3:**
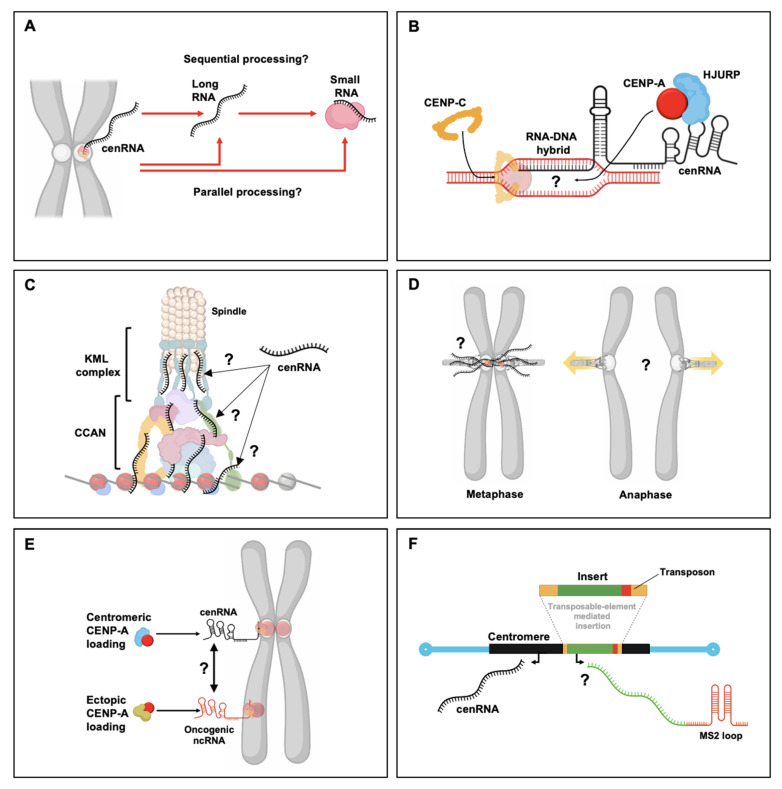
Open questions about the functional consequences of centromeric transcription. (**A**) Even though two different types of RNAs are produced from the centromere, namely, lncRNAs and small RNAs, it remains unclear whether these two types of RNAs are produced sequentially or in parallel. (**B**) Both CENP-A and CENP-B associate with centromeric transcripts and both CENP-A loading and CENP-C loading have been linked to RNA–DNA triplex formation. Additionally, in maize, CENP-C binding to centromeric DNA is associated with small RNA, whereas in humans CENP-A loading has been tied to lncRNAs. It remains unclear whether the same transcript can recruit and stabilize both CENP-A and CENP-C at centromeric chromatin. (**C**) As CENP-A and CENP-C loading is cenRNA-dependent, one intriguing question is whether the kinetochore structure as a whole is dependent on the presence of centromeric transcripts. If so, which kinetochore components drive this dependence? (**D**) The localization and activity of CPC at the inner centromere are dependent on centromeric transcripts. It will be of great interest to understand how mitotic pulling forces modulate centromeric transcription, especially during the transition from metaphase to anaphase. (**E**) CENP-A nucleosomes have been found both at the centromere and ectopically. Especially in cancers, ectopic CENP-A accumulates. Little sequence conservation exists between ectopic CENP-A sites and the centromere. This leaves the possibility that specific secondary and tertiary RNA structures exist that are found similar to/in common between transcripts derived from the centromere and ectopic sites, allowing ectopic sites to hijack CENP-A recruitment. In addition, this might also provide critical insights into the evolution of centromere DNA from a functional perspective as a CENP-A recruitment motif. (**F**) Whereas various loci on the chromosome arms have been extensively studied using genetics tools such as LacO operons and MS2/PP7 stem-loops, the centromere has been recalcitrant to genetic manipulations because of its highly repetitive nature. In *Arabidopsis thaliana*, the *A. lyrate* Tal1 LTR retrotransposon specifically integrates into the *A. thaliana* centromere. This opens the door to finally genetically modify the centromere to be able to study transcription in an inducible and tractable manner, and recruit proteins of interest.

Several big questions still await answering—not only how well conserved these functions are, but more importantly, how centromeric transcripts precisely perform their functions. What attracts centromeres and neocentromeres to be adjacent to heterochromatin? Interestingly, it is paradoxical that transcriptionally silent heterochromatin requires transcription to be formed. Is heterochromatin-associated transcription linked to the loading of new CENP-A nucleosomes? Thus, an obvious question is whether lncRNAs and small RNAs are produced from the same transcript (Figure 3A). If this were the case, this would allow for precise spatiotemporal regulation of centromeric RNAs. One primary function of centromeric transcripts appears to be loading of both CENP-A and CENP-C, setting the stage to form a functional kinetochore. RNA–DNA triplexes have been implicated in loading both proteins, thereby unraveling the role of RNA–DNA triplexes in both CENP-A and CENP-C loading (Figure 3B). Whether these loading events are synergistic is important for understanding how CENP-A chromatin is formed and how it spreads. Indeed, tantalizing evidence shows a positive feedback loop between CENP-C^CID^, dCENP-C, and CAL1 [184]. Subsequently, whether RNA has a structural role in the formation and/or maintenance of kinetochore is also an unexplored question (Figure 3C). It would be intriguing if different RNases could be linked to different kinetochore components, allowing the study of the structural role of RNA at the kinetochore. At the inner centromere, CPC is localized and activated through centromeric transcription during mitosis. Another unanswered question is whether centromeric transcription is sensitive to mitotic pulling forces (Figure 3D), potentially linking mitotic biomechanical events to direct transcriptional outputs [182]. In cancers, ectopic CENP-A nucleosomes are commonly found, yet it remains unclear how precisely CENP-A is recruited to these sites. One interesting possibility is that ectopic sites can mimic structural features of centromeric RNA, thereby hijacking CENP-A away from the centromere (Figure 3E). This could also have potential evolutionary implications if RNA folding motifs are a driving force in the potential of a sequence to become a centromere. Finally, the holy grail in studying centromeres is the ability to be able to genetically manipulation centromeres. Being able to recruit components to the centromere at will, and visualize and track transcriptional activity, have not been possible because of the centromere’s highly repetitive nature. One possibility might be to utilize the *Tal1* LTR retrotransposon from *Arabidopsis lyrate* (Figure 3F). This LTR is absent in *A. thaliana*, yet when introduced, it is still specifically incorporated at *A. thaliana’s* centromeres [185]. If this system could be engineered to study centromere biology, including centromeric transcription, a range of previously untestable hypotheses could finally be tested. The recent publication of the first telomere-to-telomere sequence of a human chromosome [186,187] might even make human centromeres a target for such studies.

All in all, the centromere transcription field is still in its early days, as many critical questions remain unanswered. Being able to learn how a fast-evolving sequence can still perform such a broad range of essential functions is especially critical for understanding how the genome integrity is regulated.

## Figures and Tables

**Table 1:** Summary of centromeric transcription as reported in various eukaryotic species, including the name of the transcribed sequence, where in the cell cycle transcription occurs, the reported lengths of the transcriptional products, any proteins they are known to interact with, whether these transcripts are 5′ capped (5′), polyadenylated (pA), and/or spliced (S), and whether these sequences have been described to act cis or trans.

Species	Sequence Name	Cell Cycle	Transcript Length	Interacting Proteins	5′	pA	S	*cis*/*trans*	Ref.
Humans (*Homo sapiens*)	α-satellite *, L1 *	early G1, mitosis	0.5–2.45 kb	CENP-A, CENP-B, CENP-C, HJURP, SGO1, Aurora B, DHX38, SUV39H1		+		*cis*	[21,22,23,24,25,26,27,28,29,30,31,32,33]
Mouse (*Mus musculus*)	minor satellite *, major satellite ^#^	S, G2/M	0.1–5 kb, 120 nt	CENP-A, Aurora B, Survivin, INCENP, WDHD1, Dicer, SUV39H2		+			[34,35,36,37,38,39,40,41,42,43,44]
Tammar wallaby (*Macropus eugenii*)	KERV-1 ^#^, sat23 ^#^		34–42 nt	CENP-B					[45,46]
*Xenopus laevis*, *Xenopus tropicalis*	cen-RNA *, fcr1 *			CENP-C, Aurora B, INCENP, Borealin			+	*trans*	[47,48,49]
Red flour beetle (*Tribolium castaneum*)	TCAST *^, #^		21–26 nt				+		[50]
Fruit fly (*Drosophila melanogaster*)	satellite III *	mitosis	~1.3 kb	CENP-C				*trans*	[51,52,53]
Budding yeast (*Saccharomyces cerevisiae*)	cenRNA *	S phase	462–1754 nt			+		*trans*	[54,55,56]
Fission yeast (*Schizosaccharomyces pombe*)	Otr ^#^, imrl ^#^, cnt *	S phase	0.5–10 kb	Rdp1, Ago1, Chp1, Clr4, Swi6	+	+	+	*cis*	[57,58,59,60,61,62,63,64,65,66,67,68]
Corn (*Zea mays*)	CentC *, CRM *		40–900 nt	CENP-A, CENP-C			+		[69,70,71,72]
*Arabidopsis thaliana*	cen180 *		24 nt						[73]
Rice (*Oryza sativa*)	CentO *, CRR *		4–15 kb; 21–25 nt			+	+		[74,75]
Sugar beet (*β vulgaris*)	pBV *, pEV satellite *		24 nt						[76]
Melon (*Cucumis melo*)	CmSat162 *, CmSat189 *								[77]
*Plasmodium falciparum*	Cen2 *, Cen3 *		75 nt, 175 nt						[78]

* centromere core region and ^#^ pericentromeric region. The yellow, blue, green, and red colors represent the Animalia, Fungi, Plantae, and Chromista kingdoms, respectively.
